# Protocol for a systematic review and network meta-analysis of the management of new onset atrial fibrillation in critically unwell adult patients

**DOI:** 10.1186/s13643-019-1149-7

**Published:** 2019-10-28

**Authors:** Brian W. Johnston, Ruaraidh Hill, Rui Duarte, Chung Shen Chean, Danny F. McAuley, Bronagh Blackwood, Nathan Pace, Ingeborg D. Welters

**Affiliations:** 1 University of Liverpool and The Royal Liverpool and Broadgreen University Hospitals, Liverpool Health Partners, Liverpool, UK; 2 Wellcome-Wolfson Institute of Experimental Medicine, Queen’s University Belfast, Belfast, UK; 3 University of Utah, Salt Lake City, USA

**Keywords:** Atrial fibrillation, Intensive care, Systematic review, Network meta-analysis

## Abstract

**Background:**

New onset atrial fibrillation is the most commonly encountered arrhythmia in critically unwell patients with a reported incidence of 4% to 29%. The occurrence of new onset atrial fibrillation may precipitate acute heart failure and lead to thromboembolic complications as well as being associated with increased in-hospital and in intensive care unit (ICU) mortality. Despite being common, much of our current knowledge regarding the treatment of new onset atrial fibrillation comes from patients with chronic atrial fibrillation or post cardiac surgery. It is unclear if management strategies in these patient cohorts can be applied to new onset atrial fibrillation in the general ICU. This protocol for a systematic review and network meta-analysis aims to address this uncertainty and define what is the most effective management strategy for the treatment of new onset atrial fibrillation (NOAF) in acutely unwell adult patients.

**Methods:**

In this systematic review and network meta-analysis, we plan to search electronic databases (Cochrane Central Register of Controlled Trials [CENTRAL], MEDLINE, EMBASE, Science Citation Index Expanded on Web of Science and relevant trial registries) for relevant randomised and non-randomised trials. Citations will be reviewed by title, abstract and full text by two independent reviewers and disagreement resolved by discussion and a third independent reviewer, if necessary. The Cochrane Risk of Bias tool will be used to assess risk of bias in randomised trials and the Risk of Bias in Nonrandomised Studies of Interventions (ROBINS-I) tool will be used for non-randomised studies. Statistical analysis will be carried out using R package meta and netmeta. We will first conduct a pairwise meta-analysis. If conditions for indirect comparison are satisfied and suitable data are available, we will conduct network meta-analysis using frequentist methodology. Treatments will be ranked according to efficacy with associated P-scores. We will assess the quality of the evidence in the pairwise using GRADE methodology and network meta-analysis comparisons in the CINeMA module in R package meta.

**Discussion:**

Our review will be the first to assess direct and indirect evidence to assess the efficacy and rank the treatments available for new onset atrial fibrillation in critically unwell patients. Our review findings will be applicable to the care of people in a range of acute settings including, ICU, the emergency department and acute medical units.

**Systematic review registration:**

PROSPERO registry number: CRD42019121739.

## Review question

What is the most effective management strategy for the treatment of new onset atrial fibrillation (NOAF) in acutely unwell adult patients?

## Background

### Description of the condition

Atrial fibrillation (AF) is one of the most commonly encountered arrhythmias in critically unwell patients. It has been reported that AF complicates approximately 4% to 29% of all intensive care unit (ICU) admissions [1–4]. Despite the high risk of AF in acute illness, much of our current knowledge comes from post-cardiac and thoracic surgical patients and may not be representative of patients admitted to a general non-cardiac intensive care unit. Moreover, guidelines for the management of AF are largely based upon guidelines for patients with chronic AF [5].

The development of AF is associated with increased length of hospital stay, increased ICU length of stay and increased mortality [3, 4, 6–8]. Klouwenberg et al. reported an increase in median length of ICU stay of 3.5 days for patients admitted with sepsis that developed NOAF compared to those without AF, and an increase in crude mortality from 14 to 29% [2]. Walkey et al. reported similar increases in ICU mortality from 37.7 to 56.3% in patients admitted with severe sepsis that developed NOAF [8]. Interestingly, in a large retrospective study, Walkey observed an increased mortality in patients that developed NOAF that was not present in those with existing AF. This suggests that NOAF in the context of severe sepsis may have an aetiology different to chronic AF [8].

Shaver et al., in one of the largest prospective studies of patients admitted to a general ICU, investigated the factors associated with NOAF compared to chronic AF [6]. It was revealed that AF was independently associated with an increased mortality regardless of whether the AF was new or chronic [6]. Chronic AF was associated with congestive cardiac failure, hypertension and hyperlipidaemia whilst NOAF was more likely to be associated with increasing age, hypotension, number of organs failing and increased vasopressor use [6]. The finding that NOAF is rarely associated with known predictors of chronic AF has been reported in other studies and adds further evidence to the suggestion that NOAF is a separate clinical entity to chronic AF [4, 6, 8, 9]. In a systematic review of the epidemiology of NOAF, Yoshida et al. highlighted the association between severity of illness as measured by Acute Physiology, Age, Chronic Health Evaluation II (APACHE II), Simplified Acute Physiology Score II (SAPS II), systemic inflammatory response syndrome (SIRS) criteria and the presence of sepsis and suggested that the presence of systemic inflammation may have a role in triggering AF in intensive care patients [4]. This is supported by evidence from prospective studies that revealed a higher median C-reactive protein (CRP) level at the time of onset of AF and a continuous increase in CRP levels in those with NOAF [1, 4]. Similar studies following coronary artery bypass surgery found that the pre-emptive use of non-steroidal anti-inflammatory drugs or colchicine was able to reduce the risk of postoperative AF [10]. Taken together, these findings further suggest that NOAF may have an inflammatory origin and unique aetiology.

### Description of the intervention

In the UK, treatment for NOAF is based upon the National Institute for Health and Care Excellence (NICE) guidelines for the management of AF, whilst in the USA, treatment recommendations are based upon the American College of Cardiology/American Heart Association (AHA/ACC) guidelines [5] [11]. These were recently updated in 2019 to reflect new evidence regarding anticoagulation of patients with AF and AF management in patients with acute coronary syndromes [12]. NICE and AHA/ACC guidelines recommend that all patients with haemodynamic compromise are treated with electrical cardioversion and rate or rhythm control pharmacotherapy in haemodynamically stable patients [5, 13]. In those patients treated with rate or rhythm control, NICE recommend that rate control only is advisable if the AF has persisted for longer than 48 h [5]. This approach likely reflects the results of large trials such as the AFFIRM (Atrial Fibrillation Follow-up Investigation in Rhythm Management) and RACE (Rate Control versus Electrical Cardioversion for Persistent Atrial Fibrillation) trial that reported more favourable outcomes in rate control strategy and fewer thromboembolic complications with rate control respectively [14, 15]. NICE recommend rate or rhythm control with Flecanide or Amiodarone but do not advocate treatment with magnesium or calcium channel antagonist medication [5]. In contrast, the AHA/ACC guidelines advocate the use of non-dihydropyridine calcium channel antagonists and beta antagonists as one of the first-line treatments to achieve rate control [11].

Despite NICE guidance, a recent UK survey revealed that 23.8% of intensive care physicians would treat NOAF with electrolyte replacement (e.g. Magnesium) as first line and add additional antiarrhythmic medications if required [5, 16]. Moreover, the survey revealed that 34.8% of physicians would treat NOAF regardless of blood pressure and heart rate, whilst 39.5% would only intervene if heart rate was between 120 and 139 bpm [16]. Amiodarone appears to be the most commonly used pharmacotherapy for NOAF with over 80% of ICU physicians using it first line [16]. Beta antagonists were the most commonly reported second-line therapy whilst medications such as calcium channel antagonists, Flecanide and Digoxin were reported less often [16]. In contrast, studies from the USA reports that calcium channel antagonists are the most commonly used anti-arrhythmic in septic critically unwell patients followed by beta antagonists, Digoxin and finally Amiodarone [17].

It is clear that there is variation in practice internationally and among ITU physicians. It has also been reported that there is a perception among physicians that a rhythm control strategy for NOAF may carry a higher risk of adverse events [18]. Despite this, a significant proportion of patients treated with rate control actually achieve cardioversion to sinus rhythm with beta antagonists having the highest rate of cardioversion to sinus rhythm of the antiarrhythmics [17, 19]. Walkey et al. revealed that beta antagonist use was associated with reduced hospital mortality compared with calcium channel antagonists, Digoxin or Amiodarone and adds to the evidence that the optimal antiarrhythmic management strategy in critically unwell patients remains unknown [16, 17, 20].

### Why is this review required?

AF affects a significant number of critically unwell patients. Despite this, there is a lack of evidence to guide how this arrhythmia should be managed. Current guidelines are based largely upon the management of chronic AF, and there is considerable variation between guidelines in the UK and the USA. Furthermore, evidence from a recent survey revealed significant variation in the UK in management of AF among ICU physicians [16]. With increasing evidence that the aetiology of NOAF in critically unwell patients may be different to chronic AF, and the detrimental effects of NOAF, there is an urgency to define the best management strategy for NOAF in acutely unwell adult patients.

### Review aims

The aims of this review are to:

1. Estimate the effectiveness of the currently used methods for treatment of NOAF in critically unwell adult patients.
2. To provide a ranking of the available treatment methods according to their effectiveness at rate or rhythm control of AF.

#### Review question and objectives

This review will address the following review question:

What is the most effective management strategy for the treatment of new onset atrial fibrillation (NOAF) in acutely unwell adult patients?

The primary objective of the systematic review is therefore to determine which anti-arrhythmic strategies result in rate control (defined as heart rate below 110 bpm) or rhythm control in acute NOAF. We have selected heart rate below 110 or rhythm control as our primary outcome measure as it is the most common clinical goal of anti-arrhythmic therapy in the management of AF.

Secondary objectives of the review include exploring evidence on (1) recurrence of AF after treatment with antiarrhythmic agents or cardioversion, (2) any adverse events following treatment with anti-arrhythmic agents (e.g. thromboembolic events) or cardioversion, (3) mortality associated with developing NOAF, (4) length of ICU admission and (5) length hospital admission.

## Methods

### Criteria for including studies in this review

This systematic review and network meta-analysis will be conducted in accordance with The Cochrane Collaboration principles of Systematic Reviews and reported following the Preferred Reporting Items for Systematic Reviews and Meta-Analyses (PRISMA) guidelines and the PRISMA Network Meta-Analysis Extension statement [21, 22].

The protocol for this study will be registered with the International Prospective Register of Systematic Reviews (PROSPERO) database (registration number: CRD42019121739).

### Type of studies

Quantitative studies will be assessed for inclusion. We will include all prospective and retrospective studies referencing adults given anti-arrhythmic medication, rate control medication or electrical cardioversion for the management of NOAF in acute illness. Randomised controlled trials, quasi-randomised studies and observational studies will be included.

### Type of participants

Papers that include adults admitted to a critical care setting (ICU, HDU, Emergency Department, Acute medical unit) who have developed or develop NOAF, including paroxysmal AF (rhythm classification by continuous electrocardiograph (ECG) monitoring or 12 lead ECG) will be included.

Papers that include the following and which cannot be disaggregated from the data of interest will be excluded:

- Patients younger than 18 years
- Pregnant women
- Any patient with known AF or a history of previous episodes of AF
- All patients who have undergone or planning to undergo cardiac surgery, permanent pacemaker insertion or surgical ablation
- Patients post cardiac/thoracic surgery

### Type of interventions and comparator(s)

Eligible studies will include any of the following interventions/treatments plus standard care:

- Any anti-arrhythmic or rate control medication (including, but not limited to):
  - Any beta antagonists
  - Any calcium channel antagonists
  - Digoxin
  - Amiodarone
  - Magnesium (given at any dose)
  - Any combination of anti-arrhythmic medications
- DC cardioversion as per advanced life support guidance [23]
- Any combination of the above interventions

### Comparator(s)

For comparative studies, any of the interventions above or placebo plus standard care.

#### Type of outcome measure(s)

##### Primary outcomes

Primary outcome measures include:

- Achievement of heart rhythm control/cardioversion to sinus rhythm
- Achievement of heart rate control (defined as heart rate less than 110 bpm)

##### Secondary outcomes

Secondary outcomes include:

- Development of permanent atrial fibrillation
- Development of recurrent paroxysmal atrial fibrillation that terminates within 48 h as defined by the European Society of Cardiology [24]
- Any thromboembolic events (such as stroke, pulmonary embolism, deep vein thrombosis, left atrial thrombus) during critical care admission
- Development of major bleeding events
- Administration of therapeutic anticoagulation as recommended in NICE guidelines [5]
- Any complication documented secondary to the intervention
- Last reported mortality
- ICU mortality
- Length of stay in critical care
- Length of hospital stay

### Search methods of identification of studies

#### Search strategy

We will identify all relevant studies regardless of publication status (published, unpublished, in press and ongoing). Where possible, individual patient data will be sourced via communication with trialists.

We will focus our search strategy on the population (acutely unwell/critically unwell) and the interventions (anti-arrhythmic/rate control medication or cardioversion) components of our review question. It will not be limited by outcome studied in order to broaden the scope of eligible studies.

A comprehensive search strategy will be constructed and implemented with the help of a health information specialist with experience in developing searches for systematic reviews.

Subject index terms (such as MeSH in MEDLINE) will be used to capture the principle elements of the systematic review question. These will be used with selected free text terms.

#### Database searches

We will search the following databases using the search terms described in Additional file 1: Cochrane Central Register of Controlled Trials (CENTRAL); MEDLINE; Embase (OVID); the Science Citation Index Expanded, accessed via Web of Science.

Identified studies published since 1990 will be included. We will also search the following ongoing trial registers to identify ongoing studies: the *meta*Register of Controlled trials (www.controlled-trials.com); the US National Institutes of Health Register (www.clinicaltrials.gov); and the World Health Organisation (WHO) International Clinical Trials Registry platform (ICTRP) (www.who.int/trialsearch).

#### Searching other resources

A review of the grey literature will be conducted using ‘Google Scholar’ and Web of Science databases. We will check relevant conference proceedings and include any potentially relevant studies. We will include guidelines and conference proceedings from the American Society of Chest Physicians, American Thoracic Society, Society of Critical Care, European Society of Intensive Care Medicine, Intensive Care Society and the European Society of Cardiology. We will also check the reference lists of all included trials for other potentially relevant studies, and review authors’ personal collections.

We will update our searches within 6 months of publication of the resulting review; we will check our citations in the protocol and update as necessary and cross check with our authors.

### Data collection and analysis

#### Citation management and screening

Citations for all studies identified during the search strategy will be exported to EndNote. All citations will be imported into Covidence systematic review platform (Veritas Health Innovation, Melbourne, Australia). Duplicate citations will be removed using the automated features of EndNote and Covidence before screening.

We will retrieve full text papers that are not excluded after title and abstract screening. Full text articles will be reviewed against inclusion and exclusion criteria by two independent reviewers. Study authors will be contacted in cases where further information is required to make a screening decision (where reports are unclear or conference abstracts from ongoing studies). Differences will be resolved through discussion, and a consensus decision reached. If necessary, a third reviewer will be involved.

Title, abstract and full text screening will be performed in duplicate by two independent reviewers. Consistency between reviewers will be assessed using the kappa statistic in Covidence with a kappa statistic > 0.6 (moderate agreement) being accepted. Any discrepancies will be resolved through discussion. A third independent senior reviewer will be consulted in any instances in which a consensus decision cannot be reached.

Primary reason for exclusion will be documented in Covidence and the screening process will be documented in a PRISMA flowchart.

#### Data extraction and management

Data will be extracted from studies following full text screening. A custom data extraction form will be generated in Covidence. The data extraction form will be piloted on 10% of included studies. Data will be extracted by two independent reviewers. Consistency between reviewers will be assessed using the kappa statistic in Covidence. A kappa statistic of > 0.6 (moderate agreement) will be required prior to full data extraction on all included studies.

The data extraction form will be designed to capture information regarding:

- *Study design*/*methodology* (title, authors, journal, publication date, study type, study period, number of participants, country, setting, inclusion/exclusion criteria.
- *Study population characteristics* (age, sex, admission diagnosis, comorbidities, setting, organ failure, severity of illness as per SOFA/APACHEII/SAPSII/SIRS criteria)
- *Interventions* (anti-arrhythmic (any), cardioversion, anti-coagulation)
- *Primary outcome* (heart rate control (< 110 bpm) and heart rhythm control))
- *Secondary outcomes*

Any inconsistencies will be resolved through discussion. Where necessary a third senior reviewer will be consulted.

If clarification is necessary, we will attempt to contact the authors for further information. Where insufficient data are presented, we will request additional information from the study authors. If data cannot be obtained from authors, the impact will be discussed as a limitation of the review.

### Risk of bias

#### Assessment of risk of bias

Reviewers will assess the methodological quality and risk of bias of all included studies and resolve any disagreements by discussion. Unresolved disagreements will be resolved through discussion with a third senior reviewer.

Randomised controlled trials:

Included randomised controlled trials will be assessed using the Cochrane Collaborations Risk of Bias (RoB) tool using the criteria outlined in the Cochrane Handbook of Systematic Reviews of Interventions (version 5) [25]. The following six domains will be assessed using RoB:

### Selection bias

- Random sequence generation (describes the method to generate the allocation sequence in enough detail to allow an assessment of whether it should produce comparable groups).
- Allocation sequence concealment (describes the methods used to conceal the allocation sequence in enough detail to allow determination as to whether intervention allocations could have been foreseen before or during enrolment).

### Performance bias

- Blinding of participants and personnel (describes the measures used, if any, to blind study participants and personnel from knowledge of which intervention a participant received. Provided information relating to whether the intended blinding was effective).

### Detection bias

- Blinding of outcome assessment (describes measures, if any, used to blind outcome assessors from knowledge of which intervention a participant received. Provides any information relating to whether the intended blinding was effective).

### Attrition bias

- Incomplete data outcome (describes the completeness of the outcome data for each main outcome, including attrition and exclusion from analysis. State whether attrition and exclusions were reported, the numbers in each intervention group, reasons for attrition/exclusions where reported, and any re-inclusions in analyses performed).

### Reporting bias

- Selective outcome reporting (states how the possibility of selective outcome reporting was examined and what was found).

### Other bias

- Additional sources of bias

We will assign a judgement regarding the risk of bias in the above domains as high, low or unclear, with unclear risk indicating either a lack of information or uncertainty over the potential for bias. Where there is a lack of detail to assign a risk of bias, we will contact corresponding trial authors for clarification.

We will construct a risk of bias table to present the results of the risk of bias assessment and perform sensitivity analyses based upon methodological quality [25].

Non-randomised controlled trials:

The methodological quality of non-randomised studies will be assessed using the ROBINS-I tool for assessing risk of bias [26].

Non-randomised trials will be assessed in the following seven domains:

### Pre-intervention

- Bias due to confounding (assesses baseline confounding that occurs when one or more prognostic variable, also predicts the intervention received at baseline)
- Bias in selection of participants into the study (bias that occurs when exclusion of some eligible participants, or the initial follow-up time of some participants, or some outcome events is related to both intervention and outcome, as there will be an association between interventions and outcome even if the effects of the interventions are identical).

### At intervention

- Bias in classification of the interventions (bias introduced by either differential or non-differential misclassification of intervention status).

### Post-intervention

- Bias due to deviations from intended interventions (bias that arises when there are systematic differences between experimental intervention and comparator groups in the care provided, which represent a deviation from the intended intervention).
- Bias due to missing data (bias that arises when later follow-up is missing for individuals initially included and followed).
- Bias in the measurement of outcomes (bias introduced by either differential or non-differential errors in measurement of outcome data).
- Bias in selection of the reported result (selective reporting of results in a way that depends on the findings and prevents the estimate from being included in a meta-analysis).

The risk of bias in non-randomised trials by categorising the risk of bias as low risk, moderate risk, serious risk or critical risk based upon their responses to signalling questions as part of the ROBINS-I tool. Where insufficient detail is reported to permit a judgement regarding the risk of bias, a no information category will be assigned, and trial authors will be contacted for clarification. Judgement regarding the risk of bias will be assigned within each domain and across domains. Where possible, the magnitude and direction of biases will be assessed and reported. We will construct a risk of bias table to present the results of the ROBINS-I assessment.

### Analysis plan

#### Measures of treatment effect

A description of all included studies will be reported in evidence tables and discussed in the text [27]. We will report participant characteristics, interventions, clinical outcomes and methodological quality. We will follow a sequential approach to data synthesis and will consider RCTs first, followed by prospective and retrospective non-randomised data.

#### Primary outcomes

Achieving rate and/or rhythm control will be reported as dichotomous data and criteria used for achieving control in the studies will be noted. We will record the number of participants experiencing the outcome and the number analysed in each group. Where reported or were data permit these to be calculated, we will present odds ratios (OR) with 95% confidence intervals.

#### Secondary outcomes

Secondary outcomes will be reported using a mix of dichotomous data and continuous data. We will present dichotomous data as number of participants experiencing the outcome and OR with 95% confidence intervals. For continuous outcome data, we will extract arithmetic means and standard deviations with 95% confidence intervals for each outcome together with the numbers analysed in each group. We will also extract medians and ranges where provided.

#### Exploratory analysis (pairwise meta-analysis)

If two or more randomised controlled trials that satisfy the inclusion criteria are available and report the same outcomes in a comparable population, we will conduct a pairwise meta-analysis using a random effects model which will be carried out for each treatment. Suitability for meta-analysis will be determined by the degree of clinical and methodological heterogeneity observed between studies.

Meta-analysis will be conducted using a frequentist random effects inverse variance analysis using the R package meta. Dichotomous outcomes will be presented using the pooled odds ratios (ORs), with 95% confidence interval (95% CI). Mean difference (MD) with 95% CI will be presented for continuous outcomes.

Statistical heterogeneity within pairwise comparisons will be assessed using the *I*^2^ measure. A high degree of heterogeneity is *I*^2^ measure > 75 [25].

Effect sizes of individual studies and any pooled estimates of effect will be presented in tables and graphically as forest plots.

#### Network meta-analysis

In addition to pairwise meta-analysis, we will assess if sufficient clinical and statistical data is retrieved to proceed to a network meta-analysis (NMA).

It is likely that a number of relevant studies will be observational in nature. We will inspect studies carefully to assess for design limitations and follow a sequential approach, considering randomised trials first followed by non-randomised trials.

We will assess the homogeneity of the evidence base and if it satisfies the assumption of transitivity, that is, that the distribution of the effect modifiers are comparable across treatments, in deciding whether to progress to a network meta-analysis [28].

If network meta-analysis is conducted, we will use a frequentist approach and a random effects model for binomial and continuous outcomes assessing the effect estimate of each treatment for AF [29].

Mean difference and OR ratios with 95% confidence and predictive intervals will be presented [29]. We will estimate and explore any orderings of treatment hierarchy in key measures of effect and present treatment rankings with P-score using Netrank of the R package netmeta [30].

Following unadjusted analysis, secondary analyses will be conducted to account for any imbalance in the distribution of effect modifiers and between study differences. Network meta-regression methods will be conducted to account for these differences [25].

Data will be formatted in contrast-based rows as specified for data input with the R packages meta and netmeta [30].

Inconsistency in the data will be assessed by fitting inconsistency model scatterplots and using Cochran’s *Q* test.

We will engage the services of a statistician with experience in systematic review, meta-analysis and network meta-analysis to advise on and assist with the statistical analysis.

### Assessment of inconsistency

#### Assessment of heterogeneity

Assessment of heterogeneity for pairwise meta-analysis will be undertaken, considering statistical as well as semi-subjective methods along with an appreciation of the included studies objectives, population, design and conduct. The degree of statistical heterogeneity will be assessed using the *I*^2^ measure, with values greater than 75% indicating a high degree of heterogeneity [25]. If the network meta-analysis contains closed loops, a formal test of consistency will be conducted. If the test of consistency is upheld, it suggests that the treatment effect from direct evidence is consistent with the treatment effect from indirect evidence [28].

#### Risk of bias across studies

##### Sensitivity analysis

To test the robustness of our data, we will conduct a sensitivity analysis for all studies assessed as being low risk of bias included in the systematic review. If potential indications of study inconsistency are encountered in the network meta-analysis, we will explore study characteristics and explore sensitivity analyses.

##### Analysis of subgroups or subset

To assess the impact of covariates and areas of heterogeneity in our sample, we will explore subgroup analysis and or use meta-regression.

Proposed covariates for subgroup analysis will include:

- Severity of illness graded by SOFA and/or APACHE score
- Location: ICU vs non-IC, HDU
- Medical vs non-medical (surgical)
- Sepsis/non-sepsis/cardiac
- Gender distribution (% males vs. % females)

##### Quality of evidence

To assess the quality of treatment effect estimates from NMA requires best estimates from direct, indirect and mixed (combined direct plus indirect) evidence, as well as quality ratings for the direct and indirect comparisons.

We will assess the quality of the evidence by following the steps to assess the quality of treatment effect estimates for network meta-analysis as per the GRADE working group recommendations [31]. We will consider each of the five components (study limitations, inconsistency, indirectness, imprecision and publication bias) of GRADE separately, before assigning a confidence in each pairwise effect size and a confidence in ranking of treatments [32].

As we expect to have a large number of interventions, we will assess the quality of evidence across the network meta-analysis will be assessed in the CINeMA web application using the R package meta as described by Salanti et al. [32].

We will (1) present direct and indirect treatment estimates for each comparison of the evidence network. The direct estimate of effect is provided by a head-to-head comparison (trials of A v B), and the indirect estimate is provided by two or more head-to-head comparisons that share a common comparator (for example, we infer the effects of A v B from trials of A v C and trials of B v C). (2) Rate the quality of each direct and indirect effect estimate. (3) Present the NMA estimate for each comparison of the evidence network. (4) Rate the quality of each NMA effect estimate [31].

## Discussion

This systematic review and network meta-analysis will synthesise the available direct and indirect evidence of the effectiveness of anti-arrhythmic medications used in the management of NOAF in critically unwell patients. NOAF in these patients is associated with an increased length of ICU and hospital stay and increased mortality and morbidity. NOAF in critical illness is increasingly recognised as a condition that has unique clinical aetiology distinct from that of patients with chronic AF. Despite this current treatment, recommendations largely come from studies in patients with chronic AF, in which there is variation of practice, both internationally and among clinicians. To our knowledge, this is the first systematic review considering treatment of AF in the acutely/critically ill patient cohort and will provide clinicians and policy makers with information on which to make evidence-based recommendations. The results of our network meta-analysis have the potential to influence the management of a large number of patients as atrial fibrillation occurs in up to 29% of patients admitted to ICU. Furthermore, our findings will likely be applicable to patients admitted to other acute care areas such as the emergency department and acute medical admissions unit.

From preliminary literature searches, it is unclear if there are many existing systematic reviews in this area and it may be that we have many more non-randomised trials. Therefore, we will thoroughly assess the risk of bias in these studies prior to progressing to NMA to ensure validity of the findings. Conducting an NMA will allow us to address more clinically relevant questions by comparing all clinically relevant comparators from the direct and indirect evidence. We will also investigate the complications and outcomes associated of NOAF.

In network meta-analysis, a network consisting of ‘nodes’ representing individual treatments and ‘lines’ connecting nodes representing direct and indirect comparisons between treatments can be drawn [33]. The amount of evidence can also be represented by the node size and line thickness. This visual representation provides a greater visualisation of the evidence with consideration of the network geometry serving to identify key areas for future research and provide a framework for conducting further clinical trials in this area [33].

We will publish the results of this review in clinical speciality journals and we will aim to present it in international meetings and conferences. We will also make our report available via our web-based repository and update our Prospero entry with the web address details.

## Supplementary information

**Additional file 1.** Medline and Embase search strategies

## Data Availability

Not applicable.

## Abbreviations

AF: Atrial fibrillation
APACHE II: Acute Physiology, Age, Chronic Health Evaluation II
CRP: C-reactive protein
ECG: Electrocardiograph
HDU: High dependency unit
ICU: Intensive care unit
NICE: National Institute for Health and Care Excellence
NMA: Network meta-analysis
NOAF: New onset atrial fibrillation
PRISMA: Preferred Reporting Items for Systematic Reviews and Meta-analyses Protocol
SAPS: Simplified Acute Physiology Score
SIRS: Systemic inflammatory response syndrome
SOFA: Sequential organ failure assessment
