# Supplementary material for: Protocol for a systematic review and network meta-analysis of the management of new onset atrial fibrillation in critically unwell adult patients
Source: Syst Rev. 2019 Oct 28;8:242. doi: 10.1186/s13643-019-1149-7 (PMC6816145; doi:10.1186/s13643-019-1149-7)
Supplement: Supplementary file 1 — Additional file 1. Medline and Embase search strategies [file 13643_2019_1149_MOESM1_ESM.docx]

**Additional file 1: Medline and Embase search strategies**

| **Databases** | **Date searched** | **No. retrieved** |
| --- | --- | --- |
| MEDLINE (Ovid), Epub ahead of print and MEDLINE In-Process (Ovid) | 11/12/2018 | 662 |
| EMBASE (Ovid) | 11/12/2018 | 1298 |

**Search strategies**

| **Database: Medline** |
| --- |
| Strategy used:   \| 1 \| Atrial Fibrillation/ \| 48328 \| \| --- \| --- \| --- \| \| 2 \| (Atrial* adj2 (Fibrillat* or flutter)).tw. \| 63328 \| \| 3 \| AF.tw. \| 34240 \| \| 4 \| (tachycardia or tachyarrhythmia or arrhythmia or supraventricular).tw. \| 87656 \| \| 5 \| 1 or 2 or 3 or 4 \| 160116 \| \| 6 \| ((new* or recent*) adj1 diagno*).tw. \| 63298 \| \| 7 \| (onset* or new*).tw. \| 3048950 \| \| 8 \| 6 or 7 \| 3053023 \| \| 9 \| Critical Care/ \| 47996 \| \| 10 \| Critical Illness/ \| 24797 \| \| 11 \| ((critical* or intensiv*) adj4 (care or ill*)).tw. \| 176778 \| \| 12 \| ((critical* or intensiv*) adj4 (care or ill* or unwell*)).tw. \| 176812 \| \| 13 \| 9 or 10 or 11 or 12 \| 198998 \| \| 14 \| 5 and 8 and 13 \| 662 \| |

| **Database: Embase** |
| --- |
| Strategy used:   \| 1 \| Atrial Fibrillation/ \| 41928 \| \| --- \| --- \| --- \| \| 2 \| (Atrial* adj2 (Fibrillat* or flutter)).tw. \| 109441 \| \| 3 \| AF.tw. \| 65045 \| \| 4 \| (tachycardia or tachyarrhythmia or arrhythmia or supraventricular).tw. \| 121780 \| \| 5 \| 1 or 2 or 3 or 4 \| 240819 \| \| 6 \| ((new* or recent*) adj1 diagno*).tw. \| 107104 \| \| 7 \| (onset* or new*).tw. \| 3689424 \| \| 8 \| 6 or 7 \| 3695973 \| \| 9 \| Critical Care/ \| 88512 \| \| 10 \| Critical Illness/ \| 26890 \| \| 11 \| ((critical* or intensiv*) adj4 (care or ill*)).tw. \| 254629 \| \| 12 \| ((critical* or intensiv*) adj4 (care or ill* or unwell*)).tw. \| 254703 \| \| 13 \| 9 or 10 or 11 or 12 \| 295857 \| \| 14 \| 5 and 8 and 13 \| 1399 \| \| 15 \| limit 14 to medline \| 91 \| \| 16 \| 14 not 15 \| 1308 \| \| 17 \| remove duplicates from 16 \| 1298 \| |
